# 5,6-Dimethyl-2-(5-methyl­thio­phen-2-yl)-1-[(5-methyl­thio­phen-2-yl)meth­yl]-1*H*-benzimidazole

**DOI:** 10.1107/S1600536814006333

**Published:** 2014-03-29

**Authors:** David K. Geiger, Ava L. Isaac

**Affiliations:** a Department of Chemistry, State University of New York-College at Geneseo, 1 College Circle, Geneseo, NY 14454, USA

## Abstract

The title mol­ecule, C_20_H_20_N_2_S_2_, is T-shaped and consists of a nearly flat 5,6-dimethyl-2-(5-methyl­thio­phen-2-yl)benzimidazole system approximately perpendicular to the 5-methyl­thio­phen-2-ylmethyl substituent. The 5,6-dimethyl-2-(5-meth­yl­thio­phen-2-yl)benzimidazole system is rotationally disordered about the two imidazole N atoms as approximated by a twofold rotation axis with a refined major/minor occupancy ratio of 0.884 (2):0.116 (2). The benzimidazole ring system is essentially planar, the largest deviations being 0.026 (2) and 0.044 (18) Å in the major and minor components, respectively. The inter­planar angles between the benzimidazole unit and the 5-methyl­thio­phen-2-yl substituent are 10.8 (3) and 8(3)° in the major and minor components, respectively, and the corresponding angles with the 5-methyl­thio­phen-2-ylmethyl substituent are 88.12 (8) and 89.5 (4)°. In the crystal, mol­ecules are oriented with their 2-(5-methyl­thio­phen-2-yl)benzimidazole mean planes approximately parallel to (11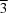) and appear to be held together by π–π [2-thiophene⋯imidazole centroid–centroid distance = 4.1383 (7) Å] and C—H⋯π contacts. A weak C—H⋯N hydrogen bond generates infinite chains parallel to [100].

## Related literature   

For the structure of 5,6-di­methyl­benzimidazole, see: Lee & Scheidt (1986 ▶). For the structure of 2-(thio­phen-2-yl)-1-(thio­phen-2-ylmeth­yl)-1*H*-benzimidazole, see: Geiger *et al.* (2012 ▶). For the 5-chloro derivative, see: Geiger & Nellist (2013*a* ▶), the 6-chloro derivative, see: Geiger & Nellist (2013*b* ▶) and the 6-bromo derivative, see: Geiger & Destefano (2012 ▶). Reich *et al.* (2004 ▶) provide examples of benzimidazole synthesis *via* inter­molecular aldimine coupling. For a discussion of the biological activity of benzimidazole derivatives, see: López-Rodríguez *et al.* (1999 ▶); Horton *et al.* (2003 ▶).
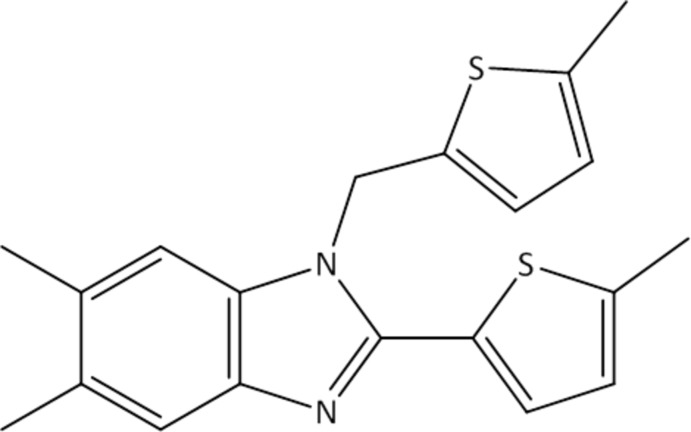


## Experimental   

### 

#### Crystal data  

C_20_H_20_N_2_S_2_
*M* *_r_* = 352.50
Triclinic, 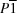

*a* = 6.4453 (11) Å
*b* = 10.0228 (18) Å
*c* = 14.249 (3) Å
α = 79.171 (5)°
β = 83.694 (5)°
γ = 83.625 (6)°
*V* = 894.8 (3) Å^3^
*Z* = 2
Mo *K*α radiation
μ = 0.30 mm^−1^
*T* = 200 K
0.40 × 0.40 × 0.40 mm

#### Data collection  

Bruker SMART X2S benchtop diffractometer
Absorption correction: multi-scan (*SADABS*; Bruker, 2013 ▶) *T*_min_ = 0.60, *T*_max_ = 0.89
10964 measured reflections
3121 independent reflections
2420 reflections with *I* > 2σ(*I*)
*R*_int_ = 0.037

#### Refinement  

*R*[*F* ^2^ > 2σ(*F* ^2^)] = 0.043
*wR*(*F* ^2^) = 0.123
*S* = 1.03
3121 reflections
276 parameters
47 restraints
H-atom parameters constrained
Δρ_max_ = 0.26 e Å^−3^
Δρ_min_ = −0.30 e Å^−3^

### 

Data collection: *APEX2* (Bruker, 2013 ▶); cell refinement: *SAINT* (Bruker, 2013 ▶); data reduction: *SAINT*; program(s) used to solve structure: *SHELXS97* (Sheldrick, 2008 ▶); program(s) used to refine structure: *SHELXL2013* (Sheldrick, 2008 ▶); molecular graphics: *PLATON* (Spek, 2009 ▶); software used to prepare material for publication: *publCIF* (Westrip, 2010 ▶).

## Supplementary Material

Crystal structure: contains datablock(s) global, I. DOI: 10.1107/S1600536814006333/qk2065sup1.cif

Structure factors: contains datablock(s) I. DOI: 10.1107/S1600536814006333/qk2065Isup2.hkl

Supporting information file. DOI: 10.1107/S1600536814006333/qk2065Isup3.mol

Supporting information file. DOI: 10.1107/S1600536814006333/qk2065Isup4.cml

CCDC reference: 993009

Additional supporting information:  crystallographic information; 3D view; checkCIF report

## Figures and Tables

**Table 1:** Hydrogen-bond geometry (Å, °)

*D*—H⋯*A*	*D*—H	H⋯*A*	*D*⋯*A*	*D*—H⋯*A*
C12—H12*A*⋯N2^i^	0.99	2.48	3.195 (4)	129
C61—H61*B*⋯C3^ii^	0.98	2.88	3.793 (8)	155
C71—H71*B*⋯C4^iii^	0.98	2.89	3.815 (4)	157

Symmetry codes: (i) 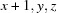; (ii) 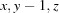; (iii) 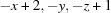.

**Fig. 1.**
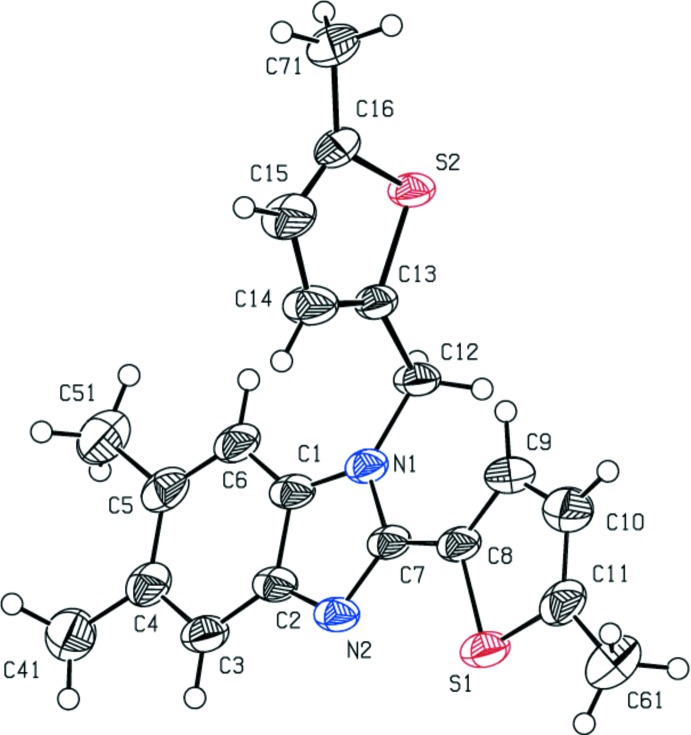
Perspective view of the title compound showing the atom-labeling scheme. Anisotropic displacement parameters are displayed at the 50% probability level. Only the major contributor to the disorder model is shown.

**Fig. 2.**
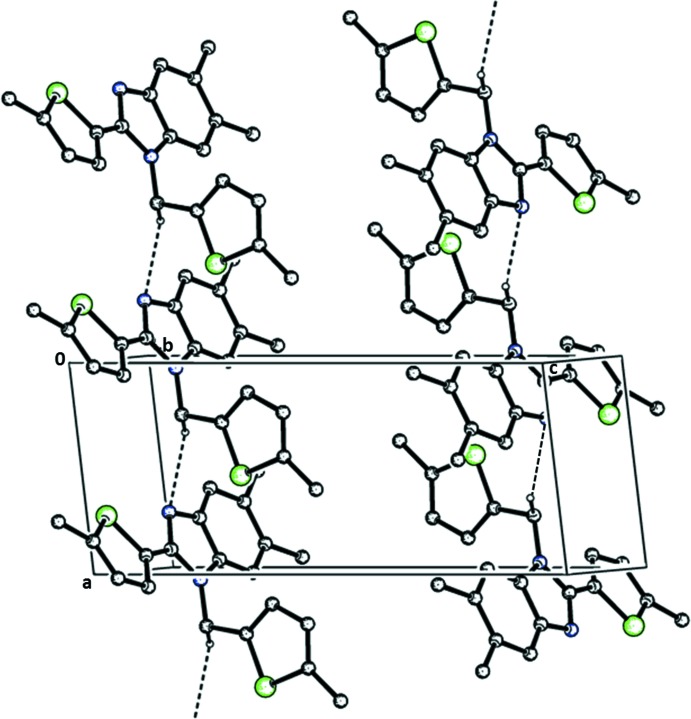
Packing diagram showing the hydrogen bonding network forming infinite chains parallel to [100]. All hydrogen atoms except H12A have been omitted for clarity. C12—H12A···N2 hydrogen bonds are represented by dashed lines. Only the major contributor to the disorder model is shown.

## supplementary crystallographic information

### 1. Comment

Benzimidazole derivatives have numerous pharmacological uses. Examples include inhibitors of serotonin activated neurotransmission drugs (López-Rodríguez *et al.*, 1999) and anti­arrhythmic, anti­histamine, anti­ulcer, anti­cancer, fungicidal, and anthelmintical drugs (Horton *et al.*, 2003). The title compound was prepared as part of our efforts to characterize benzimidazole analogues with thio­phene substitutents (Geiger & Nellist, 2013*a*; Geiger & Nellist, 2013*b*; Geiger & Destefano, 2012; Geiger *et al.*, 2012).

The title compound crystallizes with one molecule in the asymmetric unit. A perspective view of the molecule with the atom-labeling scheme showing only the the major contributor of the orientation disordered 5,6-di­methyl-2-(5-methyl­thio­phen-2-yl)benzimidazole system is given in Fig. 1. The benzimidazole ring system is essentially planar. The largest deviation from planarity is 0.0257 (22) Å for C7 and 0.044 (18) Å for N201 in the major and minor components, respectively. The 2-(5-methyl­thio­phen-2-yl) plane is canted 10.8 (3)° and 8.2 (2.6)° to the benzimidazole plane in the major and minor components, respectively.

The crystal structure reveals molecules oriented with their 2-(5-methyl­thio­phen-2-yl)benzimidazole mean planes approximately parallel to (113). Pairs of molecules related by crystallographic inversion centers exhibit π-π inter­actions. The separation between the mean planes of the associated 2-(5-methyl­thio­phen-2-yl)benzimidazole moieties is 3.74 Å with the closest C···C inter­action between symmetry-related C8 thio­phene atoms [C8···C8(2-*x*,-*y*,-*z*) = 3.727 (6) Å]. The closest C—H···C nonbonded contact is C61—H61C···C1(2-*x*,-*y*,-*z*) [H···C 2.87 Å, C···C 3.744 (5) Å]. Other C—H···π inter­actions between the methyl groups of the thio­phene substituents and the benzene ring on adjacent molecules involve C61—H61B···C3(*x*,*y*-1,*z*) [H···C 2.88 Å, C···C 3.793 (8) Å] and C71—H71B···C4(2-*x*,-*y*,1-*z*) [H···C 2.90 Å, C···C 3.815 (4) Å]. The extended structure exhibits chains formed by very weak inter­molecular C—H···N hydrogen bonds involving one of the methyl­ene hydrogen atoms (H12A) and the unsubstituted benzimidazole nitro­gen atom (N2) along the *a* axis. The result is infinite *C*(5) chains. Figure 2 displays a packing diagram exhibiting the chains parallel to [100]. The C12···N2(*x*+1,*y*,*z*) non-bonded contact is 3.195 (4) Å and the C12—H12A···N2 angle is 128.9°.

### 2. Experimental

The title compound was prepared by stirring 4,5-di­methyl-1,2-di­amino­benzene (0.200 g, 1.47 mmol) in absolute ethanol (15 ml) for five minutes under nitro­gen. 5-methyl-2-thio­phene­carboxaldehyde (0.32 ml, 0.374 g, 2.97 mmol) was added dropwise and the reaction mixture was stirred for three days at ambient temperature. The product precipitated during this time and the yellow solid was isolated by vacuum filtration and dried. The isolated yield was 0.468 g (90.4%). ^1^H NMR (400 MHz, acetone-*d*_6_, p.p.m.): 2.34 (*s*, 3H), 2.36 (*s*, 3H), 2.37 (*s*, 3H), 2.53 (*s*, 3H), 5.74(*s*, 2H), 6.24 (*d*, 1H), 6.78, (*d*, 1H), 6.87 (*d*, 1H), 7.32 (*s*, 1H), 7.39 (*d*, 1H), 7.40 (*a*, 1H). ^13^C NMR (acetone-*d*_6_, p.p.m.): 14.20, 14.24, 19.36, 19.68, 43.49, 110.39, 119.34, 125.03, 125.45, 126.29, 127.21, 130.84, 130.99, 131.63, 134.81, 137.54, 139.58, 141.96, 142.83, 146.42.

Crystals suitable for X-ray analysis were obtained by vapor diffusion of hexane into a concentrated chloro­form solution.

### 3. Refinement

Crystal data, data collection and structure refinement details are summarized in the crystallographic data table. The resolution of the data was limited to 0.84 Å (θ_max_ = 25.1°) because the data quality dropped of markedly at higher resolution. For the shell from 0.85 to 0.84 Å, the mean I/σ was 3.47.

During the initial stages of refinement, it became obvious that the molecule exhibited twofold rotational disorder. The disorder was successfully modeled using the metrics of the major component to define the minor component. Similarity restraints were used for the bond distances using SAME and anisotropic displacement parameters of the minor component atoms were constrained to those of the major component using EADP. The structure converged with a refined major:minor component ratio of 0.8843 (20):0.1157 (20).

All hydrogen atoms were observed in difference Fourier maps. The H atoms were refined using a riding model with a C—H distance of 0.99 Å for the methyl­ene carbon atoms, 0.98 Å for the methyl carbon atoms and 0.95 Å for the phenyl and pyridine carbon atoms. The methyl C—H hydrogen atom isotropic displacement parameters were set using the approximation *U*_iso_ = 1.5*U*_eq_. All other C—H hydrogen atom isotropic displacement parameters were set using the approximation *U*_iso_ = 1.2*U*_eq_.

### Crystal data

**Table:**

C_20_H_20_N_2_S_2_	*Z* = 2
*M**_r_* = 352.50	*F*(000) = 372
Triclinic, *P*1	*D*_x_ = 1.308 Mg m^−^^3^
*a* = 6.4453 (11) Å	Mo *K*α radiation, λ = 0.71073 Å
*b* = 10.0228 (18) Å	Cell parameters from 6329 reflections
*c* = 14.249 (3) Å	θ = 2.3–24.9°
α = 79.171 (5)°	µ = 0.30 mm^−^^1^
β = 83.694 (5)°	*T* = 200 K
γ = 83.625 (6)°	Block, yellow
*V* = 894.8 (3) Å^3^	0.40 × 0.40 × 0.40 mm

### Data collection

**Table:**

Bruker SMART X2S benchtop diffractometer	3121 independent reflections
Radiation source: XOS X-beam microfocus source	2420 reflections with *I* > 2σ(*I*)
Doubly curved silicon crystal monochromator	*R*_int_ = 0.037
ω scans	θ_max_ = 25.1°, θ_min_ = 2.8°
Absorption correction: multi-scan (*SADABS*; Bruker, 2013)	*h* = −7→7
*T*_min_ = 0.60, *T*_max_ = 0.89	*k* = −11→11
10964 measured reflections	*l* = −16→15

### Refinement

**Table:**

Refinement on *F*^2^	Primary atom site location: structure-invariant direct methods
Least-squares matrix: full	Secondary atom site location: difference Fourier map
*R*[*F*^2^ > 2σ(*F*^2^)] = 0.043	Hydrogen site location: inferred from neighbouring sites
*wR*(*F*^2^) = 0.123	H-atom parameters constrained
*S* = 1.03	*w* = 1/[σ^2^(*F*_o_^2^) + (0.0639*P*)^2^ + 0.397*P*] where *P* = (*F*_o_^2^ + 2*F*_c_^2^)/3
3121 reflections	(Δ/σ)_max_ < 0.001
276 parameters	Δρ_max_ = 0.26 e Å^−^^3^
47 restraints	Δρ_min_ = −0.30 e Å^−^^3^

### Special details

**Table:**

Geometry. All e.s.d.'s (except the e.s.d. in the dihedral angle between two l.s. planes) are estimated using the full covariance matrix. The cell e.s.d.'s are taken into account individually in the estimation of e.s.d.'s in distances, angles and torsion angles; correlations between e.s.d.'s in cell parameters are only used when they are defined by crystal symmetry. An approximate (isotropic) treatment of cell e.s.d.'s is used for estimating e.s.d.'s involving l.s. planes.
Refinement. Refinement of *F*^2^ against ALL reflections. The weighted *R*-factor *wR* and goodness of fit *S* are based on *F*^2^, conventional *R*-factors *R* are based on *F*, with *F* set to zero for negative *F*^2^. The threshold expression of *F*^2^ > σ(*F*^2^) is used only for calculating *R*-factors(gt) *etc*. and is not relevant to the choice of reflections for refinement. *R*-factors based on *F*^2^ are statistically about twice as large as those based on *F*, and *R*- factors based on ALL data will be even larger.

### Fractional atomic coordinates and isotropic or equivalent isotropic displacement parameters (Å^2^)

**Table:**

	*x*	*y*	*z*	*U*_iso_*/*U*_eq_	Occ. (<1)
C1	0.9366 (4)	0.2289 (3)	0.2305 (2)	0.0381 (7)	0.884 (2)
C2	0.7398 (4)	0.2498 (3)	0.19474 (18)	0.0373 (6)	0.884 (2)
C3	0.6040 (8)	0.3653 (6)	0.2051 (5)	0.0429 (12)	0.884 (2)
H3	0.473	0.3818	0.1783	0.052*	0.884 (2)
C4	0.6713 (5)	0.4579 (3)	0.2581 (2)	0.0449 (8)	0.884 (2)
C5	0.8692 (6)	0.4337 (4)	0.2962 (3)	0.0465 (9)	0.884 (2)
C6	1.0024 (9)	0.3197 (8)	0.2824 (7)	0.0454 (8)	0.884 (2)
H6	1.1353	0.3033	0.3075	0.054*	0.884 (2)
C41	0.5284 (6)	0.5834 (4)	0.2728 (3)	0.0626 (10)	0.884 (2)
H41A	0.3988	0.584	0.2426	0.094*	0.884 (2)
H41B	0.5986	0.6651	0.2436	0.094*	0.884 (2)
H41C	0.4951	0.5827	0.3416	0.094*	0.884 (2)
C51	0.9359 (6)	0.5297 (4)	0.3547 (2)	0.0683 (10)	0.884 (2)
H51A	0.845	0.5256	0.4148	0.102*	0.884 (2)
H51B	0.9244	0.623	0.3182	0.102*	0.884 (2)
H51C	1.0815	0.5029	0.369	0.102*	0.884 (2)
N1	1.0278 (3)	0.1079 (2)	0.20560 (15)	0.0366 (6)	0.884 (2)
C7	0.8828 (4)	0.0601 (3)	0.15921 (17)	0.0358 (6)	0.884 (2)
N2	0.7102 (5)	0.1432 (3)	0.1501 (3)	0.0376 (7)	0.884 (2)
C8	0.9077 (4)	−0.0691 (3)	0.1266 (2)	0.0384 (7)	0.884 (2)
C9	1.0526 (6)	−0.1817 (4)	0.1428 (2)	0.0522 (12)	0.884 (2)
H9	1.1686	−0.1863	0.1794	0.063*	0.884 (2)
C10	1.0088 (6)	−0.2882 (4)	0.0990 (3)	0.0586 (11)	0.884 (2)
H10	1.0942	−0.3723	0.1028	0.07*	0.884 (2)
C11	0.8341 (5)	−0.2615 (4)	0.0507 (2)	0.0492 (8)	0.884 (2)
S1	0.72042 (12)	−0.10176 (9)	0.05845 (6)	0.0447 (3)	0.884 (2)
C61	0.7391 (6)	−0.3515 (4)	−0.0025 (3)	0.0652 (10)	0.884 (2)
H61C	0.8254	−0.3586	−0.0627	0.098*	0.884 (2)
H61A	0.5973	−0.3124	−0.0165	0.098*	0.884 (2)
H61B	0.7324	−0.4425	0.037	0.098*	0.884 (2)
C201	0.978 (3)	−0.058 (2)	0.1416 (17)	0.0381 (7)	0.116 (2)
C202	0.775 (3)	−0.0185 (17)	0.1147 (13)	0.0373 (6)	0.116 (2)
C203	0.653 (4)	−0.098 (3)	0.073 (2)	0.0429 (12)	0.116 (2)
H203	0.5116	−0.0714	0.0591	0.052*	0.116 (2)
C204	0.769 (4)	−0.224 (3)	0.054 (2)	0.0449 (8)	0.116 (2)
C205	0.980 (4)	−0.263 (2)	0.072 (2)	0.0465 (9)	0.116 (2)
C206	1.079 (4)	−0.178 (3)	0.116 (2)	0.0454 (8)	0.116 (2)
H206	1.2211	−0.203	0.1294	0.054*	0.116 (2)
C241	0.655 (5)	−0.313 (3)	0.005 (3)	0.0626 (10)	0.116 (2)
H24A	0.5239	−0.2625	−0.0162	0.094*	0.116 (2)
H24B	0.6226	−0.3958	0.0506	0.094*	0.116 (2)
H24C	0.7437	−0.3376	−0.0505	0.094*	0.116 (2)
C251	1.108 (4)	−0.392 (2)	0.0553 (19)	0.0683 (10)	0.116 (2)
H25A	1.2486	−0.3923	0.0761	0.102*	0.116 (2)
H25B	1.1202	−0.3962	−0.0131	0.102*	0.116 (2)
H25C	1.0405	−0.4704	0.0921	0.102*	0.116 (2)
N201	1.034 (2)	0.0443 (17)	0.1869 (13)	0.0366 (6)	0.116 (2)
C207	0.865 (3)	0.1387 (16)	0.1827 (13)	0.0358 (6)	0.116 (2)
N202	0.704 (4)	0.104 (3)	0.147 (3)	0.0376 (7)	0.116 (2)
C208	0.859 (3)	0.258 (2)	0.2257 (18)	0.0384 (7)	0.116 (2)
C209	0.999 (8)	0.305 (7)	0.277 (7)	0.0522 (12)	0.116 (2)
H209	1.1339	0.2609	0.2893	0.063*	0.116 (2)
C210	0.914 (6)	0.425 (4)	0.309 (4)	0.0586 (11)	0.116 (2)
H210	0.9855	0.4688	0.3481	0.07*	0.116 (2)
C211	0.724 (5)	0.475 (3)	0.280 (2)	0.0492 (8)	0.116 (2)
S201	0.644 (2)	0.3748 (17)	0.2127 (12)	0.0447 (3)	0.116 (2)
C261	0.592 (5)	0.606 (3)	0.295 (2)	0.0652 (10)	0.116 (2)
H26A	0.4495	0.5851	0.3204	0.098*	0.116 (2)
H26B	0.5866	0.6687	0.2331	0.098*	0.116 (2)
H26C	0.6544	0.6485	0.34	0.098*	0.116 (2)
C12	1.2465 (3)	0.0569 (3)	0.21561 (16)	0.0415 (6)	
H12A	1.3325	0.1352	0.2052	0.05*	
H12B	1.2956	0.0014	0.1655	0.05*	
C13	1.2791 (3)	−0.0285 (2)	0.31259 (15)	0.0373 (5)	
C14	1.1370 (4)	−0.0773 (3)	0.38263 (18)	0.0579 (8)	
H14	0.9901	−0.0631	0.3777	0.069*	
C15	1.2288 (4)	−0.1530 (3)	0.46544 (18)	0.0588 (8)	
H15	1.1487	−0.1949	0.521	0.071*	
C16	1.4376 (4)	−0.1594 (3)	0.45742 (16)	0.0446 (6)	
S2	1.52848 (9)	−0.07226 (7)	0.34707 (4)	0.0434 (2)	
C71	1.5901 (5)	−0.2263 (3)	0.52863 (18)	0.0596 (8)	
H71B	1.513	−0.2596	0.5902	0.089*	
H71C	1.6838	−0.1597	0.5373	0.089*	
H71A	1.673	−0.303	0.5048	0.089*	

### Atomic displacement parameters (Å^2^)

**Table:**

	*U*^11^	*U*^22^	*U*^33^	*U*^12^	*U*^13^	*U*^23^
C1	0.0329 (16)	0.0515 (18)	0.0243 (13)	−0.0065 (13)	−0.0050 (13)	0.0103 (12)
C2	0.0303 (14)	0.0493 (16)	0.0253 (13)	−0.0046 (12)	−0.0030 (11)	0.0117 (11)
C3	0.042 (3)	0.052 (2)	0.034 (2)	−0.003 (2)	−0.0122 (19)	−0.0002 (14)
C4	0.052 (2)	0.0523 (18)	0.0265 (17)	−0.0064 (15)	−0.0026 (12)	0.0042 (13)
C5	0.055 (3)	0.0557 (19)	0.0268 (19)	−0.0138 (19)	−0.0042 (16)	0.0024 (15)
C6	0.0436 (16)	0.066 (3)	0.023 (2)	−0.0082 (16)	−0.0055 (12)	0.0039 (17)
C41	0.070 (3)	0.062 (2)	0.055 (2)	0.0029 (18)	−0.0082 (17)	−0.0137 (17)
C51	0.082 (2)	0.077 (2)	0.049 (2)	−0.0076 (19)	−0.0123 (18)	−0.0167 (17)
N1	0.0297 (11)	0.0525 (16)	0.0247 (12)	−0.0061 (11)	−0.0048 (9)	0.0029 (10)
C7	0.0302 (13)	0.0514 (17)	0.0210 (13)	−0.0071 (12)	−0.0036 (10)	0.0081 (11)
N2	0.0299 (11)	0.048 (2)	0.0298 (11)	−0.0043 (13)	−0.0057 (8)	0.0071 (17)
C8	0.0327 (17)	0.0553 (17)	0.0235 (14)	−0.0060 (14)	−0.0049 (12)	0.0052 (12)
C9	0.050 (2)	0.063 (2)	0.041 (3)	0.0093 (16)	−0.0188 (19)	−0.0037 (18)
C10	0.061 (2)	0.061 (2)	0.053 (3)	0.0119 (17)	−0.0163 (17)	−0.0114 (18)
C11	0.055 (2)	0.063 (2)	0.0258 (14)	−0.0114 (15)	−0.0017 (17)	0.0022 (15)
S1	0.0416 (5)	0.0579 (5)	0.0325 (5)	−0.0058 (4)	−0.0125 (4)	0.0036 (3)
C61	0.084 (3)	0.072 (3)	0.0420 (19)	−0.015 (2)	−0.012 (2)	−0.0070 (18)
C201	0.0329 (16)	0.0515 (18)	0.0243 (13)	−0.0065 (13)	−0.0050 (13)	0.0103 (12)
C202	0.0303 (14)	0.0493 (16)	0.0253 (13)	−0.0046 (12)	−0.0030 (11)	0.0117 (11)
C203	0.042 (3)	0.052 (2)	0.034 (2)	−0.003 (2)	−0.0122 (19)	−0.0002 (14)
C204	0.052 (2)	0.0523 (18)	0.0265 (17)	−0.0064 (15)	−0.0026 (12)	0.0042 (13)
C205	0.055 (3)	0.0557 (19)	0.0268 (19)	−0.0138 (19)	−0.0042 (16)	0.0024 (15)
C206	0.0436 (16)	0.066 (3)	0.023 (2)	−0.0082 (16)	−0.0055 (12)	0.0039 (17)
C241	0.070 (3)	0.062 (2)	0.055 (2)	0.0029 (18)	−0.0082 (17)	−0.0137 (17)
C251	0.082 (2)	0.077 (2)	0.049 (2)	−0.0076 (19)	−0.0123 (18)	−0.0167 (17)
N201	0.0297 (11)	0.0525 (16)	0.0247 (12)	−0.0061 (11)	−0.0048 (9)	0.0029 (10)
C207	0.0302 (13)	0.0514 (17)	0.0210 (13)	−0.0071 (12)	−0.0036 (10)	0.0081 (11)
N202	0.0299 (11)	0.048 (2)	0.0298 (11)	−0.0043 (13)	−0.0057 (8)	0.0071 (17)
C208	0.0327 (17)	0.0553 (17)	0.0235 (14)	−0.0060 (14)	−0.0049 (12)	0.0052 (12)
C209	0.050 (2)	0.063 (2)	0.041 (3)	0.0093 (16)	−0.0188 (19)	−0.0037 (18)
C210	0.061 (2)	0.061 (2)	0.053 (3)	0.0119 (17)	−0.0163 (17)	−0.0114 (18)
C211	0.055 (2)	0.063 (2)	0.0258 (14)	−0.0114 (15)	−0.0017 (17)	0.0022 (15)
S201	0.0416 (5)	0.0579 (5)	0.0325 (5)	−0.0058 (4)	−0.0125 (4)	0.0036 (3)
C261	0.084 (3)	0.072 (3)	0.0420 (19)	−0.015 (2)	−0.012 (2)	−0.0070 (18)
C12	0.0289 (12)	0.0621 (16)	0.0289 (12)	−0.0047 (11)	−0.0071 (10)	0.0067 (11)
C13	0.0326 (12)	0.0502 (14)	0.0269 (12)	−0.0030 (10)	−0.0071 (10)	0.0011 (10)
C14	0.0376 (14)	0.091 (2)	0.0374 (14)	−0.0103 (14)	−0.0063 (12)	0.0137 (14)
C15	0.0536 (17)	0.083 (2)	0.0293 (14)	−0.0117 (15)	−0.0015 (12)	0.0181 (13)
C16	0.0531 (16)	0.0521 (15)	0.0250 (12)	−0.0016 (12)	−0.0086 (11)	0.0034 (10)
S2	0.0351 (4)	0.0610 (4)	0.0287 (3)	−0.0006 (3)	−0.0088 (2)	0.0068 (3)
C71	0.0706 (19)	0.0691 (19)	0.0331 (14)	0.0073 (15)	−0.0167 (13)	0.0052 (13)

### Geometric parameters (Å, º)

**Table:**

C1—N1	1.379 (4)	C204—C205	1.413 (17)
C1—C2	1.399 (4)	C204—C241	1.515 (18)
C1—C6	1.400 (5)	C205—C206	1.390 (19)
C2—N2	1.382 (4)	C205—C251	1.500 (18)
C2—C3	1.392 (7)	C206—H206	0.95
C3—C4	1.429 (6)	C241—H24A	0.98
C3—H3	0.95	C241—H24B	0.98
C4—C5	1.419 (4)	C241—H24C	0.98
C4—C41	1.508 (4)	C251—H25A	0.98
C5—C6	1.382 (5)	C251—H25B	0.98
C5—C51	1.509 (5)	C251—H25C	0.98
C6—H6	0.95	N201—C207	1.357 (15)
C41—H41A	0.98	N201—C12	1.498 (15)
C41—H41B	0.98	C207—N202	1.312 (16)
C41—H41C	0.98	C207—C208	1.435 (16)
C51—H51A	0.98	C208—C209	1.393 (19)
C51—H51B	0.98	C208—S201	1.716 (17)
C51—H51C	0.98	C209—C210	1.40 (2)
N1—C7	1.377 (3)	C209—H209	0.95
N1—C12	1.458 (3)	C210—C211	1.351 (18)
C7—N2	1.317 (4)	C210—H210	0.95
C7—C8	1.443 (4)	C211—C261	1.516 (18)
C8—C9	1.384 (4)	C211—S201	1.666 (17)
C8—S1	1.723 (3)	C261—H26A	0.98
C9—C10	1.402 (5)	C261—H26B	0.98
C9—H9	0.95	C261—H26C	0.98
C10—C11	1.359 (5)	C12—C13	1.505 (3)
C10—H10	0.95	C12—H12A	0.99
C11—C61	1.498 (4)	C12—H12B	0.99
C11—S1	1.703 (4)	C13—C14	1.338 (3)
C61—H61C	0.98	C13—S2	1.718 (2)
C61—H61A	0.98	C14—C15	1.426 (3)
C61—H61B	0.98	C14—H14	0.95
C201—C206	1.396 (18)	C15—C16	1.333 (4)
C201—C202	1.397 (16)	C15—H15	0.95
C201—N201	1.406 (16)	C16—C71	1.504 (3)
C202—N202	1.403 (18)	C16—S2	1.724 (2)
C202—C203	1.425 (17)	C71—H71B	0.98
C203—C204	1.450 (18)	C71—H71C	0.98
C203—H203	0.95	C71—H71A	0.98
			
N1—C1—C2	105.5 (2)	C206—C205—C251	114.9 (18)
N1—C1—C6	132.9 (3)	C204—C205—C251	127.0 (18)
C2—C1—C6	121.6 (3)	C205—C206—C201	122 (2)
N2—C2—C3	128.4 (3)	C205—C206—H206	118.9
N2—C2—C1	110.1 (2)	C201—C206—H206	118.9
C3—C2—C1	121.5 (3)	C204—C241—H24A	109.5
C2—C3—C4	116.8 (3)	C204—C241—H24B	109.5
C2—C3—H3	121.6	H24A—C241—H24B	109.5
C4—C3—H3	121.6	C204—C241—H24C	109.5
C5—C4—C3	121.2 (4)	H24A—C241—H24C	109.5
C5—C4—C41	120.0 (3)	H24B—C241—H24C	109.5
C3—C4—C41	118.8 (3)	C205—C251—H25A	109.5
C6—C5—C4	120.5 (3)	C205—C251—H25B	109.5
C6—C5—C51	118.7 (3)	H25A—C251—H25B	109.5
C4—C5—C51	120.8 (3)	C205—C251—H25C	109.5
C5—C6—C1	118.4 (4)	H25A—C251—H25C	109.5
C5—C6—H6	120.8	H25B—C251—H25C	109.5
C1—C6—H6	120.8	C207—N201—C201	104.4 (13)
C4—C41—H41A	109.5	C207—N201—C12	128.0 (13)
C4—C41—H41B	109.5	C201—N201—C12	126.7 (13)
H41A—C41—H41B	109.5	N202—C207—N201	114.7 (14)
C4—C41—H41C	109.5	N202—C207—C208	123.7 (15)
H41A—C41—H41C	109.5	N201—C207—C208	121.2 (14)
H41B—C41—H41C	109.5	C207—N202—C202	105.3 (15)
C5—C51—H51A	109.5	C209—C208—C207	133.4 (18)
C5—C51—H51B	109.5	C209—C208—S201	108.7 (15)
H51A—C51—H51B	109.5	C207—C208—S201	117.9 (15)
C5—C51—H51C	109.5	C208—C209—C210	111 (2)
H51A—C51—H51C	109.5	C208—C209—H209	124.3
H51B—C51—H51C	109.5	C210—C209—H209	124.3
C7—N1—C1	106.5 (2)	C211—C210—C209	115 (2)
C7—N1—C12	128.7 (2)	C211—C210—H210	122.5
C1—N1—C12	124.2 (2)	C209—C210—H210	122.5
N2—C7—N1	112.8 (3)	C210—C211—C261	130 (2)
N2—C7—C8	122.1 (2)	C210—C211—S201	110.0 (15)
N1—C7—C8	125.1 (2)	C261—C211—S201	120.0 (18)
C7—N2—C2	105.1 (2)	C211—S201—C208	94.6 (11)
C9—C8—C7	132.5 (3)	C211—C261—H26A	109.5
C9—C8—S1	109.7 (3)	C211—C261—H26B	109.5
C7—C8—S1	117.7 (2)	H26A—C261—H26B	109.5
C8—C9—C10	112.3 (3)	C211—C261—H26C	109.5
C8—C9—H9	123.8	H26A—C261—H26C	109.5
C10—C9—H9	123.8	H26B—C261—H26C	109.5
C11—C10—C9	114.6 (3)	N1—C12—C13	112.70 (19)
C11—C10—H10	122.7	N201—C12—C13	110.9 (7)
C9—C10—H10	122.7	N1—C12—H12A	109.1
C10—C11—C61	129.0 (3)	C13—C12—H12A	109.1
C10—C11—S1	110.1 (3)	N1—C12—H12B	109.1
C61—C11—S1	120.9 (3)	C13—C12—H12B	109.1
C11—S1—C8	93.33 (15)	H12A—C12—H12B	107.8
C11—C61—H61C	109.5	C14—C13—C12	129.4 (2)
C11—C61—H61A	109.5	C14—C13—S2	110.64 (18)
H61C—C61—H61A	109.5	C12—C13—S2	119.96 (16)
C11—C61—H61B	109.5	C13—C14—C15	113.1 (2)
H61C—C61—H61B	109.5	C13—C14—H14	123.5
H61A—C61—H61B	109.5	C15—C14—H14	123.5
C206—C201—C202	117.6 (16)	C16—C15—C14	113.6 (2)
C206—C201—N201	135.0 (17)	C16—C15—H15	123.2
C202—C201—N201	107.3 (13)	C14—C15—H15	123.2
C201—C202—N202	108.0 (13)	C15—C16—C71	129.8 (2)
C201—C202—C203	125.5 (15)	C15—C16—S2	110.34 (18)
N202—C202—C203	126.1 (15)	C71—C16—S2	119.9 (2)
C202—C203—C204	112.3 (17)	C13—S2—C16	92.37 (11)
C202—C203—H203	123.8	C16—C71—H71B	109.5
C204—C203—H203	123.8	C16—C71—H71C	109.5
C205—C204—C203	124.0 (17)	H71B—C71—H71C	109.5
C205—C204—C241	119.6 (17)	C16—C71—H71A	109.5
C203—C204—C241	116.2 (17)	H71B—C71—H71A	109.5
C206—C205—C204	118.0 (17)	H71C—C71—H71A	109.5
			
N1—C1—C2—N2	0.8 (3)	C241—C204—C205—C251	−5 (5)
C6—C1—C2—N2	−177.5 (6)	C204—C205—C206—C201	0 (5)
N1—C1—C2—C3	−178.7 (4)	C251—C205—C206—C201	−176 (3)
C6—C1—C2—C3	3.1 (7)	C202—C201—C206—C205	−5 (5)
N2—C2—C3—C4	177.7 (4)	N201—C201—C206—C205	179 (3)
C1—C2—C3—C4	−2.9 (7)	C206—C201—N201—C207	175 (3)
C2—C3—C4—C5	1.3 (8)	C202—C201—N201—C207	−1 (2)
C2—C3—C4—C41	−179.0 (4)	C206—C201—N201—C12	6 (5)
C3—C4—C5—C6	0.3 (9)	C202—C201—N201—C12	−170.8 (17)
C41—C4—C5—C6	−179.4 (7)	C201—N201—C207—N202	5 (3)
C3—C4—C5—C51	−178.0 (4)	C12—N201—C207—N202	174 (2)
C41—C4—C5—C51	2.3 (6)	C201—N201—C207—C208	177 (2)
C4—C5—C6—C1	−0.3 (12)	C12—N201—C207—C208	−13 (3)
C51—C5—C6—C1	178.1 (6)	N201—C207—N202—C202	−6 (4)
N1—C1—C6—C5	−179.0 (5)	C208—C207—N202—C202	−179 (2)
C2—C1—C6—C5	−1.4 (12)	C201—C202—N202—C207	5 (3)
C2—C1—N1—C7	−1.6 (3)	C203—C202—N202—C207	178 (3)
C6—C1—N1—C7	176.3 (7)	N202—C207—C208—C209	171 (7)
C2—C1—N1—C12	170.2 (2)	N201—C207—C208—C209	−1 (8)
C6—C1—N1—C12	−11.8 (8)	N202—C207—C208—S201	−11 (4)
C1—N1—C7—N2	2.1 (3)	N201—C207—C208—S201	177.4 (18)
C12—N1—C7—N2	−169.3 (3)	C207—C208—C209—C210	−177 (4)
C1—N1—C7—C8	−175.5 (2)	S201—C208—C209—C210	5 (9)
C12—N1—C7—C8	13.1 (4)	C208—C209—C210—C211	−3 (11)
N1—C7—N2—C2	−1.6 (4)	C209—C210—C211—C261	−176 (6)
C8—C7—N2—C2	176.1 (3)	C209—C210—C211—S201	0 (8)
C3—C2—N2—C7	179.8 (4)	C210—C211—S201—C208	3 (4)
C1—C2—N2—C7	0.5 (4)	C261—C211—S201—C208	179 (3)
N2—C7—C8—C9	−166.6 (4)	C209—C208—S201—C211	−4 (5)
N1—C7—C8—C9	10.7 (5)	C207—C208—S201—C211	177 (2)
N2—C7—C8—S1	9.6 (4)	C7—N1—C12—N201	−9.2 (15)
N1—C7—C8—S1	−173.04 (19)	C1—N1—C12—N201	−179.2 (17)
C7—C8—C9—C10	177.2 (3)	C7—N1—C12—C13	−101.5 (3)
S1—C8—C9—C10	0.8 (4)	C1—N1—C12—C13	88.5 (3)
C8—C9—C10—C11	−0.6 (5)	C207—N201—C12—N1	8.3 (10)
C9—C10—C11—C61	−178.8 (4)	C201—N201—C12—N1	176 (3)
C9—C10—C11—S1	0.2 (4)	C207—N201—C12—C13	107.7 (18)
C10—C11—S1—C8	0.2 (3)	C201—N201—C12—C13	−85 (2)
C61—C11—S1—C8	179.3 (3)	N1—C12—C13—C14	10.1 (4)
C9—C8—S1—C11	−0.6 (3)	N201—C12—C13—C14	−20.7 (7)
C7—C8—S1—C11	−177.6 (2)	N1—C12—C13—S2	−168.13 (18)
C206—C201—C202—N202	−180 (3)	N201—C12—C13—S2	161.1 (6)
N201—C201—C202—N202	−2 (3)	C12—C13—C14—C15	−179.1 (3)
C206—C201—C202—C203	8 (4)	S2—C13—C14—C15	−0.7 (3)
N201—C201—C202—C203	−175 (2)	C13—C14—C15—C16	0.6 (4)
C201—C202—C203—C204	−4 (4)	C14—C15—C16—C71	178.7 (3)
N202—C202—C203—C204	−176 (3)	C14—C15—C16—S2	−0.1 (3)
C202—C203—C204—C205	−1 (5)	C14—C13—S2—C16	0.6 (2)
C202—C203—C204—C241	−177 (3)	C12—C13—S2—C16	179.1 (2)
C203—C204—C205—C206	4 (5)	C15—C16—S2—C13	−0.3 (2)
C241—C204—C205—C206	180 (3)	C71—C16—S2—C13	−179.2 (2)
C203—C204—C205—C251	179 (3)		

### Hydrogen-bond geometry (Å, º)

**Table:**

*D*—H···*A*	*D*—H	H···*A*	*D*···*A*	*D*—H···*A*
C12—H12*A*···N2^i^	0.99	2.48	3.195 (4)	129
C71—H71*B*···S201^ii^	0.98	2.94	3.912 (17)	170
C61—H61*B*···C3^iii^	0.98	2.88	3.793 (8)	155
C71—H71*B*···C4^ii^	0.98	2.89	3.815 (4)	157

Symmetry codes: (i) *x*+1, *y*, *z*; (ii) −*x*+2, −*y*, −*z*+1; (iii) *x*, *y*−1, *z*.
